# Combining Adoptive Treg Transfer with Bone Marrow Transplantation for Transplantation Tolerance

**DOI:** 10.1007/s40472-017-0164-7

**Published:** 2017-11-04

**Authors:** Nina Pilat, Nicolas Granofszky, Thomas Wekerle

**Affiliations:** Section of Transplantation Immunology, Department of Surgery, Medical University of Vienna, Waehringer Guertel 18-20, 1090 Vienna, Austria

**Keywords:** Regulatory T cells, Bone marrow transplantation, Mixed chimerism, Treg therapy, Transplantation tolerance

## Abstract

**Purpose of Review:**

The mixed chimerism approach is an exceptionally potent strategy for the induction of donor-specific tolerance in organ transplantation and so far the only one that was demonstrated to work in the clinical setting. Regulatory T cells (Tregs) have been shown to improve chimerism induction in experimental animal models. This review summarizes the development of innovative BMT protocols using therapeutic Treg transfer for tolerance induction.

**Recent Findings:**

Treg cell therapy promotes BM engraftment in reduced conditioning protocols in both, mice and non-human primates. In mice, transfer of polyclonal recipient Tregs was sufficient to substitute cytotoxic recipient conditioning. Treg therapy prevented chronic rejection of skin and heart allografts related to tissue-specific antigen disparities, in part by promoting intragraft Treg accumulation.

**Summary:**

Adoptive Treg transfer is remarkably effective in facilitating BM engraftment in reduced-intensity protocols in mice and non-human primates. Furthermore, it promotes regulatory mechanisms that prevent chronic rejection.

## Introduction

The age-long history of organ transplantation is a story of miracles, myths, and science fiction until six decades ago, when the first successful kidney transplant was performed between identical twins [1]. Some years later, the development of the first immunosuppressive drugs paved the way for the success story of clinical organ transplantation and soon it became the treatment of choice for end-stage organ diseases. Current immunosuppressive drug regimens succeed in preventing acute rejection and improve short-time survival; however, long-term outcomes did not improve to the same degree [2, 3]. Moreover, chronic use of immunosuppressive drugs is associated with increased morbidity and mortality in transplant recipients, while these medications often fail to prevent the development of chronic rejection, which is a leading cause of graft loss [4]. Induction of donor-specific immunological tolerance would not only obviate the need for life-long immunosuppression, therefore improving patient survival, but also eliminate the risk of late graft loss due to chronic rejection and preserve overall protective immunity.

Regulatory T cells (Tregs) are not only the key mediators of self-tolerance, preventing fatal autoimmunity [5], but are also recognized to play a critical role in the induction and maintenance of tolerance after organ transplantation in spontaneously tolerant patients [6].

### Induction of Tolerance

Tolerance per se is defined by as immunological non-reactivity to a specific antigen while maintaining reactivity to others, in the field of transplantation this means “the absence of graft rejection without the (chronic) use of immunosuppressive drugs” [7]. Extensive research on the way to find the “Holy Grail” of tolerance led to the development of numerous protocols for intentional tolerance induction approaches in rodent models, including functional deletion [8], costimulation blockade [9], non-depleting antibodies [10, 11], active regulatory mechanisms including Treg expansion [12], and infusion [13] or the establishment of mixed chimeras via BMT [14]. While several of these protocols yielded excellent results in mice, most of them failed when being translated into the non-human primate (NHP) system [15•].

Although mouse models have been an invaluable tool for uncovering the basic principles of allorecognition and defined strain combinations as well as genetically modified mice allowed dissection of individual pathways in transplantation immunology, there are major limitations when it comes to translation from mice to men [16]. A major hurdle for translation of murine models in the clinic is the high frequency of alloreactive memory cells found in adult humans, in contrast to young laboratory mice which are kept under SPF conditions [17]. The use of “dirty” mice would be one step towards mimicking a highly experienced immune system which may interfere with tolerance induction [18••]. Also, the use of mouse strain combinations affects outcome as some are easier to tolerize than others [19], in particular if they lack minor antigen disparities, which is not realistic in the clinical setting and has been shown to euphemize long-term graft survival [20••, 21].

Another barrier for translation of tolerance approaches from mice to humans is the limited availability of pharmaceuticals. Blockade of the CD40:CD40L pathway using an anti-CD40L mAb (MR1) was the most successful and promising approach in murine models. It is the backbone of many non-myeloablative mixed chimerism protocols, obviating the need for global T cell depletion and thymic irradiation [22]. A clinical phase I/II trial using humanized anti-CD40L antibody BG9588 had to be ended prematurely because of severe thromboembolic complications [23]. Another humanized anti-CD40L (clone hu5C8) led to severe thromboembolic complications in NHP studies [24]. Consequently, further clinical trials were put on hold [25]. Several clones of anti-CD40 have been tested as an alternative strategy to block CD40:CD40L interactions, some of them showing promising results in the prolongation of allograft survival in NHPs [26]. One of them, namely, ASKP1240, has recently been assessed for safety and efficacy in a phase II trial in kidney transplantation (ClinicalTrials.gov Identifier: NCT01780844). Currently, next-generation anti-CD40L mAbs devoid of the Fc-mediated toxicity are under development and offer promise for clinical application [27].

Preclinical animal studies using knockout, immunodeficient, or TCR transgenic strains are definitely important for mechanistic understanding of allorecognition and signaling pathways. However, for the development of tolerance strategies, more realistic approaches using stringent strain combinations including minor antigen disparities, housing in non-SPF environment and clinically available pharmaceuticals are warranted.

### Tolerance Induction Through Mixed Chimerism

Mixed chimerism denotes a state of co-existence of donor and recipient hematopoietic cells after allogeneic BMT in a pre-conditioned recipient [28]. Induction of persistent mixed chimerism has been readily achieved in murine models using numerous different strategies, and a number of protocols with reduced recipient conditioning could be developed. However, these protocols could not be readily translated into NHPs or humans as the immunologic barrier for engraftment of allogeneic BM is higher than in rodents. While permanent multilineage mixed chimerism across MHC barriers can be easily induced in rodents, the situation in NHPs and humans is more difficult. In the clinical setting, intense recipient preconditioning leads to the development of full chimerism which is undesirable in the organ transplantation setting as associated with the risk of GVHD and immune-incompetence [29]. The outstanding difference between chimerism and other tolerance strategies is the induction of “central” tolerance via intrathymic deletion by negative selection [14]. Thus, utilizing a process critical for the maintenance of self-tolerance, the mixed chimerism approach is expected to be robust and permanent.

As current clinical protocols for HLA-mismatched BMT are not ready for widespread clinical application for non-life-threatening diseases due to the toxicity related to recipient pre-conditioning and the risk of GVHD, a lot of effort has been made to develop “safer” BMT protocols. Considering the importance of regulatory mechanisms and the potency of Tregs to suppress allo-reactivity, adoptive Treg transfer has emerged as attractive possibility that might be the key to translating murine chimerism-based tolerance protocols to the clinic.

### A Role for Active Regulatory Mechanisms

Physiological self-tolerance not only involves central deletion but also critically requires non-deletional mechanisms (e.g., regulation, anergy) in order to maintain peripheral tolerance as autoreactive clones can escape negative selection. Deficits in central or peripheral tolerance cause autoimmune disease or in terms of transplantation tolerance result in graft rejection. In protocols using myeloablative and T cell depleting conditioning regimens, non-deletional tolerance mechanisms play a minor or even dispensable role likely due to the fact that potentially allo-reactive T cells are destroyed at the time of BMT and high levels of intrathymic chimerism retain central deletion of newly developing allo-reactive T cells. Experiments employing reduced intensity BMT protocols have revealed the importance for regulatory mechanisms in the induction phase of tolerance when deletion of allo-reactive T cell clones is still incomplete [30]. Tolerance, but not chimerism, could be abrogated by early treatment with anti-CD25 suggesting an active role for CD4^+^CD25^+^ Tregs, even in the presence of hematopoietic donor cells. However, neither regulation through Tregs nor classical anergy appear crucial several months after BMT for the maintenance of tolerance in established chimeras [30]. In sharp contrast, regulatory mechanisms have been shown to be critical even late after BMT in settings devoid of cytotoxic recipient conditioning [20••, 31]. Although the creation of “space” in distinct stem cell niches by myelosuppressive treatments enhances BM engraftment, it has been shown to be dispensable and can be overcome by very high doses of BM [32, 33], modulation of the immune system by targeting apoptosis [34], or adoptive Treg transfer [35]. Chimerism levels are generally lower in protocols without myelosuppression by irradiation or cytotoxic drugs; however, tolerance appears not linked to the degree of chimerism as long as it is stable and of multilineage nature. Interestingly, chimerism protocols relying on Tregs, and other regulatory mechanisms instead of deletional mechanisms only, have been shown to be superior in the prevention of chronic rejection [20••, 36] and could alleviate GVHD [37•]. This raises the question whether protocols involving intense recipient pre-conditioning preclude or at least aggravate regulatory tolerance mechanisms or if regulatory mechanisms need to be actively induced via adoptive cell transfer or other forms of immunomodulation. However, the latter explanation seems to be more likely as full immunological tolerance was also induced after BMT in protocols using various doses of TBI (up to 7 Gy) and adoptive Treg transfer [38–41]. The use of (clinically unrealistic) minor matched strain combinations and the absence of pathohistological examination of donor grafts in earlier reports of murine mixed chimerism protocols could have led to the underestimation of the importance of active regulatory mechanisms. However, recently, there have been numerous reports on “split tolerance” in chimeras [42] and it was acknowledged that chimerism and subsequent tolerance towards antigens of hematopoietic origin does not always equal full immunological tolerance towards (tissue-specific) minor antigens. Cells of hematopoietic origin were indeed shown to induce “passive tolerance” through mechanisms including apoptosis and anergy, but active regulatory mechanisms are obviously required to control anti-donor reactivity in the same manner as they are required to maintain self-tolerance [43].

### Tolerance in the Clinics: More than Just Chimerism

The original observation that the chimerism approach is successful in the clinical setting derives from numerous case reports in which conventional BMT recipients (most of them suffering from hematological malignancies) subsequently received an organ from the same donor for unrelated treatment of organ failure [44]. In such cases, organs are accepted without (chronic) conventional immunosuppression, even in an HLA donor-recipient setting [45]. Nevertheless, to date, only three centers have prospectively tested BMT for tolerance induction in the clinic, systematically translating findings from preclinical animal models to clinical kidney transplantation: the Massachusetts General Hospital/Harvard Medical School [46–47, 48••], the Stanford University School of Medicine [49–51], and the Northwestern Memorial Hospital/University of Louisville [52–54], all of them located within the USA (recently reviewed in detail in [55]).

The MGH approach is based on decade-long studies in mice and NHPs and initially tested in patient who suffered from coincidental multiple myeloma and end-stage kidney disease, legitimating BMT and cytotoxic recipient conditioning [56–59]. The initial proof-of-principle study included seven patients who received simultaneous kidney and BMT from HLA matched donors. All of them developed chimerism; however, one case of acute GVHD and two cases of chronic GVHD have been observed. Sustained renal allograft tolerance was achieved although some cases were complicated by myeloma recurrence [59]. The second patient cohort included 10 patients suffering from end-stage renal disease without concomitant hematological disease, receiving HLA-mismatched kidney and BMT. Chimerism was initially induced in all patients but was only transient and lost after approximately 2–3 weeks with no incidence for GVHD in any of the patients. Immunosuppression could be discontinued for a prolonged period of time in seven patients; three grafts have been lost due to rejection [48••]. Importantly, in the absence of persistent chimerism, regulatory mechanisms seem to play a major role in tolerance induction in these patients, although there was also evidence for (peripheral) clonal deletion of donor-reactive T cell clones [60•]. Tregs have been shown to be enriched in peripheral blood [61••] and allograft biopsies [46] after combined kidney and BMT in tolerant patients; however, it is unclear whether this is due to clonal expansion, de novo generation in the thymus or the periphery, or selective advantage with regard to conditioning and BMT. Tregs showed no decrease in demethylation status of TSDR region which is related to stability and suppressor function; moreover, the majority of Tregs presented with memory phenotype by 2 weeks post-BMT. However, the mechanisms leading to indefinite allograft survival despite the loss of chimerism in some patients still have to be fully elucidated [62].

In contrast, in murine models, stable macrochimerism and sustained presence of discrete populations of donor APCs in the thymus is a pre-requisite for durable tolerance as it ensures intrathymic deletion of alloreactive T cells. Nevertheless, high levels of chimerism do not necessarily predict long-term allograft survival and donor T cell engraftment was shown to be critical for long-term tolerance [63]. Dissociation of hematopoietic chimerism and donor graft survival is referred to as “split tolerance” phenomenon. Although still incompletely understood, incomplete tolerance towards skin or solid organ allografts is triggered by tissue-specific antigens and does not involve direct allorecognition [64]. Studies in human renal transplantation patients and NHPs have clearly demonstrated that the situation is different and long-term allograft survival can be achieved with transient chimerism only [46, 65, 66]. Continued renal allograft survival in the absence of permanent chimerism suggests a dominant role for peripheral mechanisms in non-murine models. Whereas the liver has been uniformly recognized a “tolerogenic” organ and a sound percentage of operational tolerance was reported in liver allograft recipients, also, the kidney itself was shown to induce tolerance towards cardiac allografts in miniature swine models [67]. Likewise, in human tolerance studies, the kidney is suggested to be important for maintaining tolerance by peripheral, most likely regulatory mechanisms.

### Tregs for Tolerance Induction

Cell therapy approaches using CD4^+^CD25^+^FoxP3^+^ Tregs for tolerance induction have been envisioned since the (re)discovery of these powerful suppressor cells more than two decades ago [68] and is currently investigated by researchers all over the globe. Some of the world’s most renowned research institutes are collaboratively working on gaining insight into the immunomodulatory mechanisms, cell product development, and clinical trial management (e.g., onestudy.org).

The therapeutic potential of Tregs was underlined by numerous reports, showing potent effects in pre-clinical autoimmune and allo-transplantation models; however, in these reports, the use of lymphopenic hosts [69, 70] or TCR transgenic Tregs [71, 72] was necessary and only minor antigen disparities or single MHC mismatches could be tolerated, respectively. So far, Tregs have not been shown to induce skin graft tolerance across MHC barriers on their own in unmanipulated recipients with a polyclonal T cell repertoire. Moreover, Tregs with indirect antigen specificity are suggested to be required for the prevention of chronic allograft rejection [38, 72], exacerbating clinical implementation.

Although the hype has stagnated a little bit during the last years due to the realization of several hurdles associated with adoptive Treg therapy [73], a recent study reported on successful induction of allograft tolerance through infusion of regulatory cells in liver transplantation patients [74•]. Still, tolerance protocols relying exclusively on peripheral tolerance are in danger to be eventually overwhelmed by the continuous thymic output of donor-reactive T cells [75] or to be broken by extreme activation of the immune system, e.g., by severe infections [76] or by successive antigen challenges [77].

### Combining Chimerism and Adoptive Treg Transfer

Given the potency of both approaches, combination of the mixed chimerism approach and adoptive Treg transfer seems to be the next logical step on the search for the holy grail of tolerance [78]. The proof of principle for the success of this combined approach was already given in several studies: co-infusion of Tregs with allogeneic BM was shown to promote BM engraftment [40, 41, 79] in pre-conditioned mice and enabled the development of the first clinically relevant BMT protocol devoid of cytotoxic recipient conditioning [35]. Although several protocols for combined BMT/Treg infusions are published (see Table 1 [35, 36, 38–41, 79–84]), there are still many open questions and concerns, precluding their immediate translation into the NHP setting and the clinics. Based on the experience in the murine setting [35], the first NHP protocol for chimerism and tolerance induced by the combination of BMT and therapeutic Treg transfer has recently been developed [85••]. Treg treatment led to the induction of multilineage chimerism which lasted longer than in all the previous NHP studies and which notably included the T cell lineage. Long-term donor kidney graft survival was achieved, even in the setting of delayed kidney transplantation. This NHP study provides a proof of principle that Treg co-transfer can promote BM engraftment and prevent allograft rejection. However, early CMV reactivation interfered with chimerism induction in some animals, necessitating further refinement of the protocol for translation into the clinics.

**Table 1:** Combining BMT and adoptive Treg transfer in pre-clinical animal models

		Recipient			Treg therapy			Outcome	
References	Model	MHC type	Conditioning	BM dosing	Cell product	Generation	Dosing	Chimerism	Tolerance
Joffre 2004 [79] (Blood)	Murine	Semi mismatch (H2bd > H2b)	8.5Gy TBI, T cell cotransfer	5 × 10^6^ (semiallogeneic)	In vitro-activated recipient CD4^+^CD25^+^ Tregs	Allogeneic (donor-type APCs + IL-2)	≤ 3 × 10^6^	Durable	–
Taylor 2004 [39] (Blood)	Murine	Full mismatch	4–5Gy TBI	20 × 10^6^ TCD	In vitro-activated donor CD4^+^CD25^+^LSelHi Tregs	Polyclonal (anti-CD3/CD28 + IL-2)	(2×) 3.5 × 10^6^	Multilineage	–
Hanash 2005 [40] (Blood)	Murine	Full mismatch	7Gy TBI	0.5 × 10^6^ TCD	Freshly isolated donor CD4^+^CD25^+^ T cells	Freshly isolated	1 × 10^6^	Multilineage	–
Joffre 2008 [38] (Nat Med)	Murine	Full mismatch	5Gy TBI	10 × 10^6^ TCD	In vitro-activated recipient CD4^+^CD25^+^ Tregs	Allogeneic (donor-type APCs + IL-2)	≤ 2 × 10^6^	Multilineage	Skin/heart
Pilat 2010 [78] (AJT)	Murine	Full mismatch	No irradiation, CTLA4Ig, anti-CD154, Rapa	20 × 10^6^	In vitro-activated recipient Foxp3-transduced Tregs, nTregs and TGFβ-induced iTregs	Polyclonal (anti-CD3/CD28 + IL-2; TGFβ; FoxP3 retrovirus)	3–5 × 10^6^	Multilineage	Skin
Pilat 2011 [41] (Transplantation)	Murine	Full mismatch	1Gy TBI, CTLA4Ig, anti-CD154	20 × 10^6^	In vitro-activated recipient Foxp3-transduced Tregs	Polyclonal (anti-CD3/CD28 + IL-2; FoxP3 retrovirus)	(2×) 2 × 10^6^	Multilineage	Skin
Lin 2012 [80] (J Surg Res)	Murine (rat)	Full mismatch	No irradiation, CTLA4Ig, anti-CD154, Rapa	100 × 10^6^	In vitro-activated recipient CD4^+^CD25^+^ Tregs	Polyclonal (anti-CD3/CD28 + IL-2)	3 × 10^6^	Multilineage	VCA
Hongo 2012 [81] (Blood)	Murine	Full mismatch	2.4Gy TLI, ATS, anti-CD25	50 × 10^6^	Freshly isolated recipient CD4^+^CD25^+^ Tregs	Freshly isolated	1 × 10^6^	Multilineage	Heart
Pilat 2014 [36] (JHLT)	Murine	Full mismatch	No irradiation, CTLA4Ig, anti-CD154, Rapa	20 × 10^6^	In vitro-activated recipient iTregs	Polyclonal (anti-CD3/CD28 + IL-2 +TGFβ)	3 × 10^6^	Multilineage	Heart
Im 2014 [82] (Stem Cells Dev)	Murine	Full mismatch	1.5Gy TBI, CY	25–30 × 10^6^	Combination of in vitro activated recipient Tregs and MSCs	Polyclonal (anti-CD3/CD28 + TGFβ)	(2×) 2 × 10^6^	Multilineage	Skin
Pilat 2015 [83] (J Immunol Res)	Murine	Full mismatch	No irradiation, CTLA4Ig, anti-CD154, Rapa	20 × 10^6^	In vitro activated recipient CD4^+^CD25^+^ Tregs	Polyclonal (anti-CD3/CD28 + IL-2)	0.5–3 × 10^6^	Multilineage	Skin
Ruiz 2015 [84] (Front Immunol)	Murine	Full mismatch	3Gy TBI, CTLA4Ig, Rapa	20 × 10^6^	In vitro RA-induced recipient iTregs	Allogeneic (APCs + RA + IL-2 + TGFβ)	2.2 × 10^6^	Multilineage	Skin
Pilat 2016 [20••] (JCI)	Murine	Full mismatch	No irradiation, CTLA4Ig, anti-CD154, Rapa	20 × 10^6^	In vitro-activated recipient CD4^+^CD25^+^ Tregs	Polyclonal (anti-CD3/CD28 + IL-2)	1 × 10^6^	Multilineage	Skin/heart
Duran-Struuk 2017 [85••] (Transplantation)	NHP	MHC mismatched	1.5Gy TBI, 7Gy TBI, ATG, CsA, anti-CD154	1.3–3 × 10^8^ (/kg)	In vitro-activated autologous CD4^+^CD25^+^ Tregs	Polyclonal (APCs, anti-CD3, IL-2, irradiated donor PBMCs)	(5×) 15–53 × 10^6^	Multilineage	Kidney

*TBI* total body irradiation, *APC* antigen-presenting cell, *TCD* T cell depleted, *Rapa* rapamycin, *VCA* vascularized composite allograft, *TLI* total lymphoid irradiation, *ATS* anti-thymocyte serum, *CY* cyclophosphamide, *MSC* mesenchymal stem cells, *RA* retinoic acid, *NHP* non-human primates, *ATG* anti-thymocyte globulin, *CsA* cyclosporin, *PBMC* peripheral blood mononuclear cell

Most of the protocols with combined Treg and BMT are employing clinically achievable doses of allogeneic BM (20 × 10^6^ which corresponds to ≈ 1 × 10^9^ cells/kg). Recipient preconditioning was non-myeloablative and consisted mostly of mild doses of irradiation and/or costimulation blockade targeting the CD40:CD40L or CD28:B7 pathway. Notably, Treg transfer was capable of obviating the need for recipient irradiation (or cytotoxic drug treatment), allowing for the first time engraftment of fully mismatched BM without myelosuppression [35]. Comparable numbers of Tregs were used in all murine protocols ranging from 0.5 to 5 × 10^6^ cells. The most interesting and critical question is the source of Tregs (donor- vs recipient- vs third party-derived) and the need for specificity (polyclonal vs allo-/donor-specific Tregs). Several groups reported that antigen-specific Tregs are more potent compared to a polyclonal Treg population in models with [79] or without BMT [72, 86, 87] and that indirect specificity is critical for the prevention of chronic allograft rejection [13, 38]. However, most of these studies compared in vitro expanded alloantigen-specific Tregs which have been activated with IL-2 for several days to freshly sorted polyclonal Tregs. Indeed, whereas Treg activation is claimed to be antigen-specific, their suppressor function might not be, at least in in vitro assays [88]. This hypothesis is supported by the fact that in vitro-activated polyclonal Tregs are potent in promoting BM engraftment [35, 80] and the prevention of acute and chronic allograft rejection [36].

### Treg Conundrum: Less Chimerism, More Tolerance?

We and others could previously show that therapeutic Treg treatment leads to engraftment of clinically realistic doses of BM and sustainable tolerance in stringent strain combinations [35, 38]. Tolerance induced by Tregs was superior to other chimerism protocols, which failed to prevent chronic rejection triggered by minor antigens; moreover, tolerance could be verified in primarily vascularized cardiac allografts and highly immunogenic skingrafts likewise. Both protocols were based on different conditioning regimens (irradiation vs costimulation blockade) and used different Treg populations (in vitro-activated polyclonal vs antigen-specific), highlighting the potency of Tregs in chimerism-based tolerance strategies. Unfortunately, both protocols are not ready for immediate clinical translation as either 5 Gy total-body-irradiation or costimulation blockade with anti-CD40L was utilized, so some fine-tuning is warranted.

In our “Treg-BMT” protocol, clinically, realistic numbers of BM and Tregs were infused into the recipient under the cover of costimulation blockade and a short course of rapamycin. Treg infusion is critical for BM engraftment and could not be replaced by IL2/anti-IL2 complex-based immunomodulation [89]. Importantly, the tolerance achieved using Tregs was superior to well-established BMT protocols using recipient TBI to allow BM engraftment [21, 22, 36]. The use of fully mismatched strain combinations revealed incomplete tolerance in non-myeloablative regimens relying primarily on deletional tolerance mechanisms [21]. Chimeras induced with low-dose irradiation presented with profound histopathologic signs of chronic rejection at the end of follow-up in both skin and heart allografts, which were shown be caused by minor antigen disparities. However, in sharp contrast, grafts from Treg-induced chimeras were devoid of chronic rejection [35, 36]. Leucocyte infiltrates in the grafts of Treg-treated chimeras were enriched in FoxP3^+^ Tregs, which were shown to have an active role in the mediation of graft survival [20••, 35]. Our data suggest that adoptive Treg transfer not only allows BM engraftment and the induction of chimerism in the absence of cytotoxic recipient conditioning but prevents graft rejection mediated by minor antigens via linked suppression [20••]. Thus, regulatory mechanisms due to adoptive Treg transfer maintain tolerance towards tissue-specific minor antigens of the donor via active intragraft regulation [20••]. Therapeutic Tregs transfer in the absence of substantial cytotoxic “danger-prone” host conditioning is therefore likely to allow for creation of a tolerogenic state involving “infectious tolerance”-like mechanisms that protect allografts from chronic rejection directed towards non-MHC tissue-specific antigens.

## Conclusion

More than 60 years after Owen and Medawar’s ground-breaking work on experimental tolerance induction by creation of mixed hematopoietic chimeras, tolerance has become a clinical reality only in a few highly selected recipients of living-donor kidney transplantation. Decades of intensive research in murine, swine, and NHP models led to the acknowledgement of peripheral regulatory mechanisms as critical factor for sustained allograft survival and the prevention of chronic rejection. Although peripheral regulation of (allo)immune responses is recognized as a complex network of multiple regulatory cell types, CD4^+^CD25^+^FoxP3^+^ Tregs have emerged as the most potent and important population in the maintenance of self- and allograft tolerance. The combination of the mixed chimerism approach with therapeutic Treg cell therapy has shown great potency in preclinical animal models in terms of both safety and efficacy. Treg treatment not only enabled tremendous decrease in recipient conditioning, a prerequisite for widespread clinical application, but also created genuine tolerance inhibiting chronic rejection of allografts. We think that combining the mixed chimerism approach and adoptive Treg transfer allows for implementation of complementary tolerance mechanisms in the recipient, therefore mimicking the complex system of self-tolerance.
